# Do adolescent sedentary behavior levels predict type 2 diabetes risk in adulthood?

**DOI:** 10.1186/s12889-021-10948-w

**Published:** 2021-05-22

**Authors:** Jillian A. Scandiffio, Ian Janssen

**Affiliations:** 1 School of Kinesiology and Health Studies, Queen’s University, Kingston, Ontario K7L 3N6 Canada; 2 Department of Public Health Sciences, Queen’s University, Kingston, Canada

**Keywords:** Sedentary behavior, Type 2 diabetes, Cohort study, Adolescence

## Abstract

**Background:**

The objective was to determine whether time spent in different types of sedentary behavior during adolescence are associated with the risk of developing type 2 diabetes in adulthood.

**Methods:**

Participants were 3942 adolescents aged 16 years who were part of the 1970 British Cohort Study. Sedentary behavior was assessed using a questionnaire that asked participants to indicate how much time they spent watching TV and videos, using the computer, reading, and doing homework. Incident cases of type 2 diabetes were determined quadrennially until 46 years of age. The association between adolescent sedentary behaviors and type 2 diabetes was determined using Cox proportional hazards regression that controlled for sex, body mass index, sugary beverage consumption, smoking status, physical activity at baseline, and physical activity in adulthood .

**Results:**

There were 91 incident cases of type 2 diabetes with an incidence rate of 9 cases/10,000 person-years. By comparison to those who watched TV and videos for 2 or less hours/day, type 2 diabetes risk was not different in those who watched for 2.1–4.0 h/day (HR = 0.89, 95% CI = 0.54, 1.47) but was increased by 2.06-fold (95% CI = 1.24, 3.43) in those who watched for more than 4 h/day. Time spent using a computer, reading, and doing homework were not significantly associated with type 2 diabetes.

**Conclusion:**

Spending more than 4 h/day watching television and videos at age 16 was associated with an increased risk of type 2 diabetes. Conversely, using a computer and non-screen based sedentary behaviors were not associated with type 2 diabetes risk.

## Background

Excessive sedentary behavior, and screen time in particular, is common among children and adolescents worldwide [1]. In a 2019 summary of data from 49 countries, only 34–39% of school-aged children and adolescents met sedentary behavior guideline recommendations, which in most countries includes limiting recreational screen time to 2 h per day or less [1]. High sedentary behavior and screen time levels during adolescence are associated with risk factors for chronic diseases such as obesity, high fasting insulin levels, and the metabolic syndrome [2].

While a large body of cross-sectional research has demonstrated that screen time is associated with chronic disease risk factors during adolescence, the effects of non-screen based sedentary behaviors and the long-term implications of excessive sedentary behavior on chronic disease outcomes have not been well studied. Type 2 diabetes is one chronic disease that may be influenced by adolescent sedentary behavior. Although type 2 diabetes does not usually develop until adulthood, it is a progressive disease that is typically preceded by insulin resistance that persists over years or decades [3]. Thus, type 2 diabetes may have its origins in childhood and adolescence, when insulin resistance may start to develop.

To our knowledge, only one study has examined whether sedentary behavior in adolescence predicts the development of diabetes [4]. That study examined 3717 youth aged 11–21 years who were followed over 14 years. It found that the group defined by a clustering of low physical activity and high sedentary behavior had a 69% increased likelihood of developing diabetes by comparison to a group defined by a clustering of low physical activity and low sedentary behavior. This result suggests that high sedentary behavior in adolescence may be a risk factor for diabetes. However, that study’s assessment of sedentary behavior was limited to screen time measures and it did not examine multiple levels of sedentariness, as participants were dichotomized into high or low sedentary behavior clusters. Furthermore, the 14-year follow-up, which started at ages 14 to 21 year of age, may not have been long enough for the cohort to develop a sufficient number of diabetes cases.

Therefore, the purpose of this study was to determine whether different levels of both screen-based and non-screen based sedentary behaviors during adolescence are associated with the risk of developing type 2 diabetes in adulthood. We studied these associations using data from a longitudinal cohort, with assessments of sedentary behavior at age 16 and a subsequent 30-year follow-up for incident cases of type 2 diabetes.

## Methods

### Study design and participants

This study is based on data from the 1970 British Cohort Study, an ongoing prospective birth cohort conducted by the British Centre for Longitudinal Studies [5]. The cohort is comprised of 17,196 individuals born in the United Kingdom between April 5–11, 1970 and an additional 930 individuals born in the same week who immigrated to the United Kingdom before age 16 [5]. Data collection took place in 1970, 1975, 1980, 1986, 1996, 2000, 2004, 2008, 2012 and 2016.

Ethics approval for the 1970–1996 cycles was granted from a UK National Health Service ethics review board. Starting in the 2000 cycle, ethics approval was acquired from multicentre research ethics committees [6]. Ethics for the secondary analysis presented in this paper was obtained from the General Research Ethics Board at Queen’s University. In the 1970 cycle, consent was obtained by parents verbally agreeing to participate in the interview or by returning the questionnaire [6]. In the 1975–1986 cycles, informed parental consent was obtained [6]. In the 1996–2016 cycles, participants gave written consent [6].

This present study used the 1986 cycle, when participants were aged 16 years, as the baseline. Data collection for this cycle consisted of 16 questionnaire instruments that were sent to the participants’ schools or homes. A total of 11,622 participants responded to at least one of the 16 questionnaires. For the present study, participants were excluded from the analyses if they had diabetes at age 16 (*n* = 31), if they did not respond to the sedentary behavior questions (*n* = 7205), if they did not participate in any of the subsequent cycles of the study (*n* = 426), or if they reported that they had diabetes in one cycle and then reported that they did not have diabetes in one of the subsequent cycles (*n* = 18). This left a final sample of 3942. Compared to those who were included in the analyses, those who were excluded were more likely to be male (51% vs 44%, *p* < .0001) and a current smoker at baseline (25% vs 18%, *p* < .0001) and less likely to have a parent with a college degree (23% vs 34%, *p* < .0001).

### Measurement and categorization of sedentary behavior

The sedentary behavior questionnaire consisted of a series of questions that asked participants how much time they spent watching TV, watching videos, using the computer, reading, and doing homework. Similar questionnaires administered to youth have been shown to have fair reliability with an intraclass correlation coefficient of 0.57 for repeated surveys on time spent watching television, reading, and using the computer conducted two weeks apart [7].

In three separate items, participants were asked to indicate if they watched TV, watched videos, and used the computer for ‘none’, ‘less than 1 hour’, ‘more than 1 hour’, ‘more than 2 hours’, ‘more than 3 hours’, ‘more than 4 hours’ or ‘more than 5 hours’ per day. A time value that corresponded to the midpoint of each option was assigned as follows: 0, 30, 90, 150, 210, 270 and 330 min/day. Since watching TV and watching videos were similar behaviors, the time spent in these domains were summed into a single watching TV or videos variable. Furthermore, a total screen time variable was created by summing the time spent watching TV, watching videos, and using the computer.

In two separate items, participants indicated whether they spent ‘no time’, ‘less than 1 hour’, ‘more than 1 hour’, ‘more than 2 hours’, ‘more than 3 hours’, or ‘more than 4 hours’ per day reading and doing homework. A time value that corresponded to the midpoint of each option was assigned as follows: 0, 30, 90, 150, 210 and 270 min/day. A reading and doing homework variable was created by summing the time spent in these two sedentary behaviors.

Participants were put into groups of ‘≤120 min/day’ (meeting screen time recommendations), ‘121–240 min/day’ (not meeting screen time recommendations), and ‘> 240 min/day’ (double the screen time recommendations) based on their time spent watching TV and videos and their total screen time [8]. A ‘none’ group was not created as only a small proportion of participants had no screen time. For computer use, reading, and homework, participants were put into groups of ‘none’, ‘1–30 min/day’, ‘> 30 min/day’. These cut-points were chosen to fall on half hour time increments and to maintain adequate sample sizes in each group; very few participants responded that they spent more than 1 h/day using the computer or reading and very few spent more than 2 h/day doing homework. For combined time spent reading and doing homework, participants were put into groups of ‘none’, ‘≤120 min/day’, and ‘> 120 min/day’. Again, these cut-points were chosen to fall on meaningful time increments (i.e., hourly) and to maintain an adequate sample size in each group.

### Measurement of type 2 diabetes

Diabetes cases were self-reported. A study of British adults found that 95% of self-reported diabetes cases were confirmed with medical records, which suggests that diabetes can be self-reported accurately [9]. Pre-existing diabetes cases in 1986 (when participants were 16 years old) were determined retroactively using data from the 2000 and 2004 cycles. In these cycles, participants self-reported if they had been diagnosed with diabetes and indicated how old they were when they were first diagnosed. There were 31 pre-existing diabetes cases in 1986. These participants were removed from analysis. Incident type 2 diabetes cases during the follow-up period were determined in the 1996–2016 cycles. In 1996, participants indicated whether they had been diagnosed with diabetes. Additional questions included in the 2000 cycle asked participants to indicate whether their diabetes was insulin or non-insulin dependent. If their diabetes was described as insulin dependent (i.e., type 1), they were not considered an incident case and were censored in 1996. In the 2004–2016 cycles, participants were again asked to indicate if they had diabetes. Since type 1 diabetes is unlikely to be diagnosed past age 30, we assumed that any newly reported cases of diabetes in the 2004–2016 cycles were cases of type 2 diabetes [10].

The follow-up length for participants who were never diagnosed with diabetes was determined as the difference between 1986 and the last cycle in which they participated, with a maximum follow-up length of 30 years at which time they were 46 years old. Those who reported that they had type 2 diabetes in the 1996–2016 cycles were considered incident cases and their follow-up length was determined as the difference between 1986 and the cycle in which they first reported diabetes.

### Confounding variables

Confounding variables were selected based on their established associations with both sedentary behavior and type 2 diabetes. Both time-independent (i.e., constant in all cycles) and time-dependent (i.e., varied from cycle to cycle) covariates were included. The time-independent covariates, which were based on self- and parental-reported data from the 1986 cycle, were sex (male or female), ethnicity (white or non-white), parental education (no qualifications, O-levels, A-levels or college degree or higher), and adolescent health behaviors including fruit consumption (0–1, 2–3, 4–5, or 6–7 times per week), sugary beverage consumption (none, 1–2 glasses per day, 3–4 glasses per day, or more than 4 glasses per day), takeout food consumption (none, once per week, or twice or more per week), and adolescent physical activity (physical activity quartiles based on self-reports of the number, frequency, and intensity of moderate-to-vigorous intensity physical activities performed [11]). Adult physical activity (self-reported frequency of participating in moderate-to-vigorous intensity physical of 0 times per week, once or less per week, 2–3 days per week, 4–5 days per week, or 6–7 days per week) reflected an average score based on data collected in the 2000, 2004, 2012 and 2016 cycles and was treated as a time-independent covariate [11]. The time-dependent covariates, which were measured in every cycle, were the body mass index (BMI) category (not overweight, overweight, or obese using age-specific thresholds at age 16 [12] and adult thresholds in the later cycles [13]), smoking status (non-smoker, occasional smoker, or smoker), and personal education (none, National Vocational Qualification Level 1, 2, 3, 4, or 5). If a participant was missing time-independent covariate data, they were placed in a ‘missing’ category instead of being removed from analysis. If a participant was missing data for a time-dependent covariate, the response from the previous cycle was carried forward. If time-dependent covariate data was missing for all cycles, participants were placed in a ‘missing’ category.

### Statistical analysis

Statistical analyses were performed using SAS Version 9.4 (SAS Institute Inc.). Standard descriptive statistics, such as proportions and incidence rates, were used to describe the cohort. Chi-square tests were used to compare baseline data proportions between those who developed and those who did not develop type 2 diabetes. Cox proportional hazards regression was used to examine the relationship between the sedentary behavior variables at age 16 and the risk of developing type 2 diabetes during the follow-up period. The proportional hazards assumption was tested and held for all models. Interactions between sex and the sedentary behavior variables were tested and since none were significant, males and females were included in the same analyses. For each sedentary behavior variable, three Cox models with different covariates were fit. Model 1 was limited to the primary exposure variable, the sedentary behavior characteristic at age 16. Model 2 also included the confounding variables; a stepwise elimination using a liberal *p*-value of 0.2 was used to remove confounding variables that were not associated with the outcome. Model 3 adjusted for the other sedentary behavior characteristics in addition to the confounding variables controlled for in Model 2. Because BMI is a possible mediator between sedentary behavior and type 2 diabetes, models 2 and 3 were re-run without BMI. Since this did not change the hazard ratio (HR) estimates for the sedentary behavior variables, BMI was retained as a confounding variable. Finally, we explore the continuous relationship between the sedentary behavior variables and type 2 diabetes risk using restricted cubic spline Cox proportional hazard regression. Knots were placed at the 5th, 25th, 50th, 75th, and 95th percentile of the sedentary behavior variables. Cubic spline smoothing was used to join the segments. The same confounding variables used in Model 3 for the categorical analysis were used in the restricted cubic spline regression.

## Results

Descriptive characteristics of the 3942 participants included in the final analyses are displayed in Table 1. Slightly more than half were female (55.9%) and non-smokers (56.1%) and the vast majority were Caucasian (74.9%). There were significant differences in baseline BMI category (*p* < .0001), parental education (*p* = 0.02), fruit consumption (*p* = 0.04), and sugary beverage consumption (*p* = 0.02) between those who developed and did not develop type 2 diabetes during follow-up.

**Table 1:** Descriptive characteristics of participants at baseline in 1986

	All participants (*n* = 3942)	Participants who developed type 2 diabetes (*n* = 91)	Participants who did not develop type 2 diabetes (*n* = 3851)	*P* value for diabetes vs. non-diabetes
Sex				0.06
Male	1738 (44.1)	49 (53.9)	1689 (43.9)	
Female	2204 (55.9)	42 (46.1)	2162 (56.1)	
Missing	0	0	0	
Ethnicity				0.39
White	2953 (74.9)	66 (72.5)	2887 (75.0)	
Other	115 (2.9)	1 (1.1)	114 (3.0)	
Missing	874 (22.2)	24 (26.4)	850 (22.1)	
BMI category				< 0.0001
Not overweight	1900 (48.2)	21 (23.1)	1879 (48.8)	
Overweight	247 (6.3)	19 (20.9)	228 (5.9)	
Obese	43 (1.1)	6 (6.6)	37 (1.0)	
Missing	1752 (44.4)	45 (49.5)	1707 (44.3)	
Parental education				0.02
No qualifications	518 (13.1)	20 (22.0)	498 (12.9)	
O-levels	953 (24.2)	15 (16.5)	938 (24.4)	
A-levels	268 (6.8)	9 (9.9)	259 (6.7)	
Degree or higher	879 (22.3)	14 (15.4)	865 (22.5)	
Missing	1324 (33.6)	33 (36.3)	1291 (33.5)	
Smoking status				0.15
Non-smoker	2212 (56.1)	49 (53.9)	2163 (56.2)	
Occasional smoker	1005 (25.5)	19 (20.9)	986 (25.6)	
Smoker	682 (17.3)	23 (25.3)	659 (17.1)	
Missing	43 (1.1)	0	43 (1.1)	
Fruit consumption				0.04
0–1 times/week	451 (11.4)	18 (19.8)	433 (11.2)	
2–3 times/week	890 (22.6)	26 (28.6)	864 (22.4)	
4–5 times/week	871 (22.1)	15 (16.5)	856 (22.2)	
6–7 times/week	1090 (27.7)	21 (23.1)	1069 (27.8)	
Missing	640 (16.2)	11 (1.7)	629 (16.3)	
Sugary beverage consumption				0.02
None	676 (17.2)	9 (9.9)	667 (17.3)	
1–2 glasses/day	1224 (31.1)	41 (45.1)	1183 (30.7)	
3–4 glasses/ day	680 (17.3)	18 (19.8)	662 (17.2)	
> 4 glasses/day	595 (15.1)	11 (12.1)	584 (15.2)	
Missing	767 (19.5)	12 (13.2)	755 (19.6)	
Takeaway consumption				0.11
None	1545 (39.2)	34 (37.4)	1511 (39.2)	
1 time/week	1456 (36.9)	28 (30.8)	1428 (37.1)	
> 2 times/week	747 (19.0)	26 (28.6)	721 (18.7)	
Missing	194 (4.9)	3 (3.3)	191 (5.0)	
Physical activity at age 16				0.65
Quartile 1	864 (21.9)	22 (24.2)	842 (21.9)	
Quartile 2	1019 (25.9)	19 (20.9)	1000 (26.0)	
Quartile 3	1037 (26.3)	23 (25.3)	1014 (26.3)	
Quartile 4	1022 (25.9)	27 (29.7)	995 (25.8)	

There was a total of 105,690 person-years of follow-up. The average follow-up length was 26.8 years with a range of 10 to 30 years. There were 91 incident cases of type 2 diabetes with an incidence rate of 9 cases per 10,000 person-years.

Table 2 shows the incidence rates and HRs for type 2 diabetes based on screen-based sedentary behaviors at age 16. Prior to controlling for confounding variables (model 1), there was an increased risk of developing type 2 diabetes in those who watched TV and videos for > 240 min/day (HR = 2.31, 95% CI = 1.41, 3.78) but not in those who watched TV and videos for 121–240 min/day (HR = 0.99, 95% CI = 0.60, 1.63) by comparison to those who watched for ≤120 min/day. This risk estimate in the > 240 min/day group was slightly attenuated but remained significant after adjusting for the confounding variables (model 2, HR = 2.01, 95% CI = 1.22, 3.32) and other sedentary behaviors (model 3, HR = 2.06, 95% CI = 1.24, 3.43). Total screen time levels > 240 min/day were also significantly associated with an increased risk of type 2 diabetes after controlling for confounding variables and non-screen based sedentary behaviors (model 3, HR = 1.86, 95% CI = 1.13, 3.06). However, computer use on its own was not associated with type 2 diabetes risk with a fully adjusted HR of 1.21 (95% CI = 0.52, 2.84) in the group who used a computer for 1–30 min/day and 1.11 (95% CI = 0.44, 2.80) in the group who used a computer for > 30 min/day.

**Table 2:** Risk of type 2 diabetes in adulthood according to screen-based sedentary behaviors at age 16

	N	Person-years of follow-up	Number of cases of type 2 diabetes	Type 2 diabetes cases/10,000 person-years	Hazard ratio (95% confidence interval) for type 2 diabetes		
					Model 1	Model 2	Model 3
Watching TV and videos							
≤ 120 min/day	1857	49,910	36	7	1	1	1
121–240 min/day	1404	38,012	27	7	0.99 (0.60, 1.63)	0.89 (0.54, 1.47)	0.89 (0.54, 1.47)
> 240 min/day	681	17,768	28	16	2.31 (1.41, 3.78)*	2.01 (1.22, 3.32)*	2.06 (1.24, 3.43)*
Computer							
None	3606	96,920	80	8	1	1	1
1–30 min/day	197	5128	6	12	1.43 (0.63, 3.29)	1.20 (0.52, 2.81)	1.21 (0.52, 2.84)
> 30 min/day	139	3642	5	14	1.71 (0.69, 4.22)	1.26 (0.50, 3.16)	1.11 (0.44, 2.80)
Total screen time							
≤ 120 min/day	1821	48,950	36	7	1	1	1
121–240 min/day	1385	37,478	25	7	0.91 (0.55, 1.52)	0.83 (0.50, 1.39)	0.83 (0.49, 1.38)
> 240 min/day	736	19,262	30	16	2.23 (1.37, 3.62)*	1.85 (1.13, 3.04)*	1.86 (1.13, 3.06)*

Model 1 is unadjusted. Model 2 is adjusted for sex, BMI category, sugary beverage consumption, smoking status, physical activity at baseline and physical activity in adulthood. Ethnicity, parental education, fruit consumption, and takeout eating were removed during the stepwise elimination process. Model 3 is adjusted for Model 2 covariates plus the other sedentary behavior variables (e.g., watching TV and videos was adjusted for computer, reading, and homework)

*P < 0.05

**Fig. 1:**
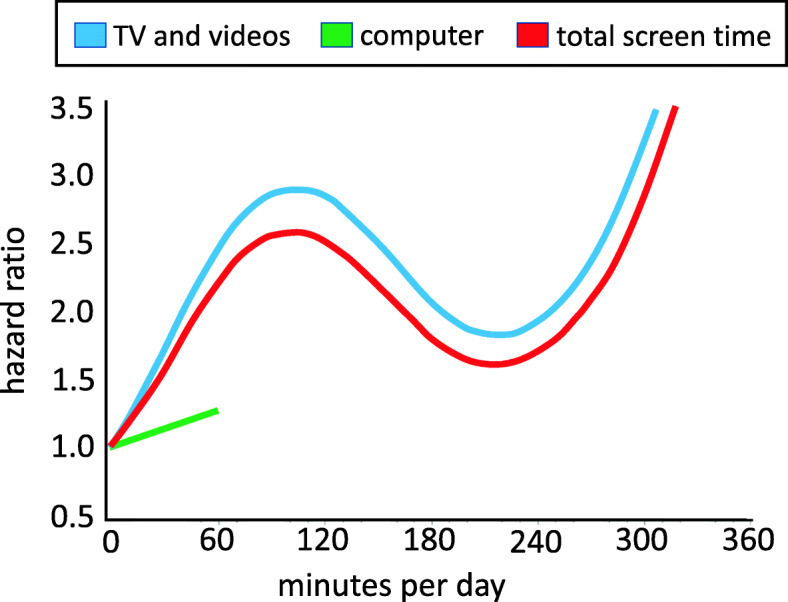
Hazard ratio for type 2 diabetes in adulthood based on continuous screen time measures (in minutes per day) obtained at age 16. Time spent watching TV and videos is represented by the blue line, time spent using a computer is represented by the green line, and total screen time is represented by the red line

The relationship between continuous measures of the screen time variables and type 2 diabetes risk, as determined from the restricted cubic spline regression and after controlling for confounding variables and other sedentary behaviour variables, is illustrated in Fig. 1. The *p* values for the test for overall significance of the curve were .02 for watching TV and videos, 0.87 for computer use, and .02 for total screen time.

Table 3 shows the HRs for type 2 diabetes based on non-screen based sedentary behaviors at age 16. Homework, reading, and the combined time spent in homework and reading were not associated with type 2 diabetes risk in any of the models. In the fully adjusted model, the HRs were 1.13 (95% CI = 0.67, 1.86) in the group who spent > 30 min/day doing homework, 1.13 (95% CI = 0.62, 2.07) in the group who spent > 30 min/day reading, and 1.24 (95% CI = 0.68, 2.27) in the group who spent > 120 min/day doing homework and reading.

**Table 3:** Risk of type 2 diabetes in adulthood according to non-screen based sedentary behaviors at age 16

	N	Person-years of follow-up	Number of cases of type 2 diabetes	Type 2 diabetes cases/10,000 person-years	Hazard ratio (95% confidence interval) for type 2 diabetes		
					Model 1	Model 2	Model 3
Homework							
None	2512	67,060	59	9	1	1	1
1–30 min/day	373	10,022	9	9	1.01 (0.50, 2.04)	1.12 (0.55, 2.26)	1.13 (0.56, 2.31)
> 30 min/day	1057	28,608	23	8	0.89 (0.55, 1.44)	1.05 (0.65, 1.72)	1.13 (0.67, 1.86)
Reading							
None	2246	59,986	47	8	1	1	1
1–30 min/day	1082	29,148	30	10	1.30 (0.82, 2.05)	1.39 (0.87, 2.20)	1.39 (0.87, 2.22)
> 30 min/day	614	16,556	14	8	1.07 (0.59, 1.94)	1.15 (0.63, 2.10)	1.13 (0.62, 2.07)
Reading and doing homework							
None	1479	39,220	31	8	1	1	1
1–120 min/day	1643	44,338	43	10	1.20 (0.75, 1.90)	1.34 (0.84, 2.14)	1.37 (0.86, 2.19)
> 120 min/day	820	22,132	17	8	0.94 (0.52, 1.70)	1.16 (0.64, 2.12)	1.24 (0.68, 2.27)

Model 1 is unadjusted. Model 2 is adjusted for sex, BMI category, sugary beverage consumption, smoking status, physical activity at baseline and physical activity in adulthood. Ethnicity, parental education, fruit consumption, and takeout eating were removed during the stepwise elimination process. Model 3 is adjusted for Model 2 covariates plus the other sedentary behavior variables (e.g., homework was adjusted for TV and videos, computer, and reading)

**Fig. 2:**
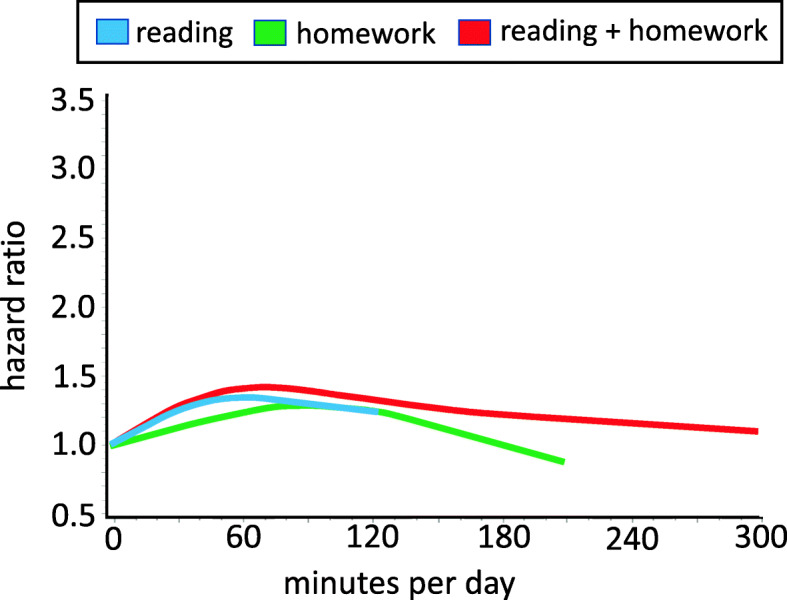
Hazard ratio for type 2 diabetes in adulthood based on continuous non-screen based sedentary behaviors (in minutes per day) obtained at age 16. Time spent reading is represented by the blue line, time spent doing homework is represented by the green line, and time spent reading and doing homework is represented by the red line

Figure 2 illustrates the relationship between continuous measures of reading and doing homework with type 2 diabetes after controlling for confounding variables and other sedentary behaviour variables. The *p* values for the test for overall significance of the curve were > 0.8 for reading, homework, and the combination of these two variables.

## Discussion

The objective of this study was to determine if time spent in different types of sedentary behavior in adolescence are associated with the risk of developing type 2 diabetes. We observed that 16-year-olds who watched TV and videos for more than 4 h/day had a twofold increased risk for developing type 2 diabetes over the next three decades. This association was independent of physical activity and several other behavioral and sociodemographic factors. Time spent using a computer, doing homework, and reading at age 16 were not associated with type 2 diabetes risk.

Our finding that adolescent screen time was associated with type 2 diabetes is consistent with a systematic review which reported that 3 out of 3 studies examining the association between screen time and a composite cardiometabolic risk score in adolescence found significant associations [2]. Another review of 8 cross-sectional studies found that high screen time was not associated with metabolic syndrome when a 2 h/day cut-point defined high screen time [14]. However, the two studies in that review that used a 5 h/day cut-point both reported that high screen time was associated with an increased odds of metabolic syndrome [15, 16]. Our findings are also consistent with two previous reviews of the adult screen time literature, both of which reported that excessive TV viewing is a risk factor for type 2 diabetes [17, 18].

To our knowledge, only one study has examined the association between adolescent sedentary behavior and diabetes risk in adulthood. That study examined screen-based sedentary behaviors and physical activity in 11–21 years old who were followed over 14 years [4]. Participants were classified into low physical activity/low sedentary behavior, low physical activity/high sedentary behavior, and high physical activity/low sedentary behavior groups. The low physical activity/high sedentary behavior group had a 69% increased likelihood of developing diabetes compared to the low physical activity/low sedentary behavior group. This is consistent with our finding that screen time in adolescence is associated with type 2 diabetes risk in adulthood independent of physical activity.

As noted in a recent systematic review, limited research has considered whether non-screen based sedentary behaviors are associated with cardiometabolic risk factors [2, 14]. One cross-sectional study found that reading was not associated with a cardiometabolic risk score, fasting serum insulin, or fasting blood glucose [19]. Several studies have compared the association with health outcomes for total sedentary time to those for screen time. A review of these studies indicates that screen time but not total sedentary time in adolescence is associated with cardiometabolic risk factors [20]. This suggests that non-screen based sedentary behaviors are not associated with cardiometabolic risk factors. This is consistent with our findings, which show that homework and reading are not associated with type 2 diabetes risk. However, it should be noted that few participants spent more than 2 h/day reading and doing homework, so it is unknown if very high levels of these sedentary behaviors influence type 2 diabetes risk. For screen time, type 2 diabetes risk was only increased in the group with values that exceeded 4 h/day. Furthermore, the lack of an association between homework and reading with type 2 diabetes risk may reflect the homogeneity of these variables as 63.7 and 57.0%, respectively, were in the “none” group for these two sedentary behavior variables. More research is needed to gain a clearer understanding of the role non-screen based sedentary behaviors have on health.

Our findings have implications for public health. They suggest that policies, programs, and interventions that aim to reduce sedentary behavior should emphasize screen-based sedentary behaviors. In addition to being an independent risk factor for type 2 diabetes, screen time is associated with other unhealthy behaviors such as increased snacking, increased consumption of sugar sweetened beverages, and decreased consumption of fruits and vegetables, [21]. Another important implication of our findings relates to the cut-point used to denote unacceptable screen time levels. Current public health recommendations in several countries are that adolescents limit recreational screen time to 2 or less hours per day [22]. We observed that the risk of diabetes was not increased in adolescents who accumulated 2–4 h/day of screen time. Based on the continuous analysis, a statistically significant increase in type 2 diabetes risk was not observed until 5 h per day of screen time. This suggests that the screen time recommendation may be overly conservative, at least when it comes to type 2 diabetes risk. However, future research is needed on a variety of health outcomes to determine the most ideal screen time cut-point for public health recommendations.

This study was not free of limitations. The self-reported study measures, including the measures of sedentary behavior and type 2 diabetes, were subject to recall bias and measurement error which likely biased the observed associations. Additionally, some potentially important confounding variables, such as family history of diabetes, were not measured or controlled for. Furthermore, it would have been ideal to control for adult sedentary behavior levels to determine if the association between adolescent sedentary behavior and type 2 diabetes risk was independent of what was done in adulthood. In addition, our study examined the risk for type 2 diabetes cases that developed at age 46 or earlier. It is possible that the relationship between adolescent sedentary behavior and diabetes cases that develop earlier in life is different from the relationship for cases that develop later in life. Another limitation, which is unavoidable in a longitudinal study of sedentary behavior, is that the adolescent sedentary behaviors, especially screen time, were considerably different at baseline (in this case in 1986) than they are today. As evidence of this, only 8.5% of the participants included in our study indicated that they spent any time on a computer in 1986. By comparison, recent statistics for the United Kingdom suggest that ~ 95% of adolescents have an internet connection at home and own a smartphone [23]. Even watching TV programs is different in 2021 than it was in the past as adolescents are not longer limited to a few TV channels that they can watch on their family TV in preprogrammed 30 or 60-min increments.

## Conclusions

There was an increased risk for type 2 diabetes in those who watched TV and videos for > 4 h/day. Computer use, reading, and doing homework were not associated with type 2 diabetes risk. These findings highlight adolescence as an important window for type 2 diabetes risk. This opens the door for future research to examine whether adolescent sedentary behaviors, especially screen time, are associated with other diseases (e.g., cardiovascular disease, cancer) that typically occur in adulthood.

## Data Availability

The data in this paper are based on data from the 1970 British Cohort Study. The data was deposited at the UK Data Service by the Centre for Longitudinal Studies at the Institute of Education, University of London. A description of all study data and information on how to apply for access to the data is provided at the following link: https://cls.ucl.ac.uk/cls-studies/1970-british-cohort-study/

## Abbreviations

CI: confidence interval
BMI: body mass index
BMI: body mass index
